# Impediment to Symbiosis Establishment between Giant Clams and Symbiodinium Algae Due to Sterilization of Seawater

**DOI:** 10.1371/journal.pone.0061156

**Published:** 2013-04-16

**Authors:** Takeo Kurihara, Hideaki Yamada, Ken Inoue, Kenji Iwai, Masayuki Hatta

**Affiliations:** 1 Ariake Yatsushiro Center, Seikai National Fisheries Research Institute, Nagasaki City, Japan; 2 Research Center for Subtropical Fisheries, Seikai National Fisheries Research Institute, Okinawa, Japan; 3 Ishigaki Branch, Okinawa Prefectural Fisheries & Ocean Research Center, Okinawa, Japan; 4 Okinawa Prefectural Sea Farming Center, Okinawa, Japan; 5 Department of Biology, Ochanomizu University, Tokyo, Japan; Leibniz Center for Tropical Marine Ecology, Germany

## Abstract

To survive the juvenile stage, giant clam juveniles need to establish a symbiotic relationship with the microalgae Symbiodinium occurring in the environment. The percentage of giant clam juveniles succeeding in symbiosis establishment (“symbiosis rate”) is often low, which is problematic for seed producers. We investigated how and why symbiosis rates vary, depending on whether giant clam seeds are continuously reared in UV treated or non treated seawater. Results repeatedly demonstrated that symbiosis rates were lower for UV treated seawater than for non treated seawater. Symbiosis rates were also lower for autoclaved seawater and 0.2-µm filtered seawater than for non treated seawater. The decreased symbiosis rates in various sterilized seawater suggest the possibility that some factors helping symbiosis establishment in natural seawater are weakened owing to sterilization. The possible factors include vitality of giant clam seeds, since additional experiments revealed that survival rates of seeds reared alone without Symbiodinium were lower in sterilized seawater than in non treated seawater. In conclusion, UV treatment of seawater was found to lead to decreased symbiosis rates, which is due possibly to some adverse effects common to the various sterilization techniques and relates to the vitality of the giant clam seeds.

## Introduction

Many animals in tropical sea areas depend on symbiosis with the zooxanthellal algae, Symbiodinium, to live in oligotrophic waters [1], [2], [3], [4]. Some species of corals, jellyfishes, anemones, sponges, polyclads, and giant clams take advantage of the symbiotic relationship in acquiring a considerable part of their necessary carbon [5], [6], [7], [8]. Most of these animals establish the symbiotic relationship through taking Symbiodinium from environmental pools, not directly from maternally derived strains [4]. Therefore, it is important for these animals to increase the probability of establishing a symbiotic relationship with Symbiodinium [9], [10], [11], [12].

The increase of the probability of symbiosis establishment is also important for giant clams from the viewpoint of the seed production [13], [14], [15]. In the tropical Indo West Pacific area giant clams are utilized as ornaments, food, and a tourism resource [13], [16]. To enhance the stock of giant clams depleted owing to overfishing and environmental destruction [14], [17], seed production has been developed in various countries [13]. In the seed production planktonic D-shaped larvae of giant clams are artificially presented with Symbiodinium [15], [16]. Some of these clams ingest the algae and propagate them to the mantle edge in the zooxanthellal tubes from Day≈15 after fertilization (“symbiosis establishment stage”, following [18]). The probability that the clams attain the symbiosis establishment stage (i.e. the number of clams establishing a symbiotic relationship/the number of all the clams reared from the larval stage; “symbiosis rate”, hereafter) is as low as <5% for Tridacna crocea Lamarck, 1819, Tridacna derasa (Röding, 1798) and Tridacna squamosa Lamarck, 1819 [19]. Most of the clams which cannot attain the symbiosis establishment fail to survive the juvenile stage [13]. This hinders efficient seed production of giant clams.

Therefore, it is necessary to examine potential factors affecting symbiosis rates of giant clams. Possible factors strongly affecting symbiosis rates include UV treatment of seawater in which giant clam seeds and Symbiodinium are reared. This is suggested from a preliminary experiment [20], which showed a decreased symbiosis rate of T. crocea seeds in UV treated seawater. The negative influence of UV treatment of seawater has, however, not been scrutinized in terms of either the reproducibility of the previous results or the mechanism that leads to the reduction of the symbiosis rate. Because seawater is frequently UV treated in seed production of marine animals [21], the possible adverse influence should be carefully examined.

Here, we investigate how and why the symbiosis rate decreases when seawater is sterilized by UV treatment and other techniques. First, we examined the reproducibility of the negative effect of UV treatment on the symbiosis rate in a hatchery (“hatchery observations”) and laboratory (“UV treatment experiment”). Second, we examined whether symbiosis rates also decrease in autoclaved and microfiltered seawater (“filtration autoclave experiments”). Because these investigations demonstrated reduced symbiosis rates in all the sterilized seawater (see Results), we further investigated the possible mechanism. That is, third, we examined whether survival rate of giant clam seeds reared without Symbiodinium is lower in sterilized seawater than in non treated seawater (“monoculture experiment”); if so, giant clam seeds are likely to be adversely affected by sterilization of seawater, thus more likely to fail attaining symbiosis establishment. Finally, we examined whether symbiosis rates in sterilized seawater increase if giant clam seeds are provided with food phytoplankton (“feeding experiment”). The hypothesis here is that sterilization of seawater might cause a decrease of giant clam food item availability including phytoplankton and thus lead to increased stress and decreased symbiosis rates for giant clam seeds. All the observations and experiments followed Fisheries Research Agency Guidelines for the experimental use of animals [22].

## Materials and Methods

### Definition of Life Stage

In this paper the term “larva” denotes a giant clam before settlement; “juvenile” a mobile clam after settlement; and “seeds” both the larva and the juvenile [12], [13]. The term “Day” denotes days from fertilization. The term “symbiosis establishment” denotes a stage such that a juvenile propagates Symbiodinium to the mantle edge in the zooxanthellal tubes on Day ≈ 15, following Hirose et al. [18].

### Preparation of Symbiodinium

We isolated Symbiodinium from the mantle of adult T. crocea, T. maxima, T. squamosa and T. derasa. Giant clam larvae were provided with either fresh or cultured Symbiodinium in the hatchery observations and with only cultured Symbiodinium in the other experiments. The culture of Symbiodinium was performed, using Daigo’s IMK Medium for Marine Microalgae (Nihon Pharmaceutical Co. Ltd.; see [20] for more details about the isolation and culture of Symbiodinium). The Symbiodinium are likely to belong to the clades A, C and D [23].

### Treatment of Seawater

We conducted hatchery observations at the Ishigaki Branch, Okinawa Prefectural Fisheries and Ocean Research Center (OPFORC; 24°27′50′′N, 124°8′35′′E) and laboratory experiments at the Ishigaki Tropical Station, Seikai National Fisheries Research Institute (SNFRI; 24°27′16′′N, 124°13′12′′E). Seawater used in the hatchery observations was pumped through a 10-µm filter from 5 m depth in the reef area near OPFORC and, when necessary, was UV treated with Funatech FL-3 (wavelength: 253.7 nm, irradiance: 35,000 µW/cm^2^)(http://www.funatech.com/sakkin_ryusui.html). Seawater used in the laboratory experiments was manually sampled from the sea surface near SNFRI and was UV treated, autoclaved, or filtered. For the UV treatment 300 ml of the seawater poured into a tray (water depth: 0.5 cm) was exposed to UV (wavelength: 253.7 nm, irradience: 9,580 µW/cm^2^) for 10 minutes with a Coralife Turbo Twist 3X UV Sterilizer (http://coralifeproducts.com/product/ultraviolet-sterilizers/). For the autoclaved treatment the sampled seawater was treated at 2 atm and 121°C for 20 minutes. For the microfiltration the sampled seawater was treated with a 0.2 µm filter. In coarser filtration the sampled seawater was treated with 0.7, 1.6, 3.0, 5.0, 11.0, and 20.0 µm filters. Some of the filtered seawater was further either UV treated or autoclaved to combine the treatments of seawater. All seawater treated was left for 0.5 to 1 day before being used to rear giant clam larvae.

The characteristics of seawater before and after disinfection are summarized in Tables S1, S2, and S3. UV treated and non treated seawater both showed a similar ozone density (≦ 0.03 ppm) day after the treatment (Table S1). Bacterial densities (colony forming units/ml) in the 0.2-µm filtered seawater were always 0 and less than the densities in the more coarsely filtered seawater (Table S2). Bacterial densities were also always 0 in the UV treated and the autoclaved seawater whereas they were 200.0±173.2 (mean ± SD) in the non treated seawater. Particulates in the fraction between 0.2 and 3.0-µm accounted for 37.2 to 56.6% of the total weight of particulates >0.2 µm in the seawater (Table S3).

### Hatchery Observations

Giant clam seeds were produced in OPFORC 61 times in total between 2007 and 2008: 17 times for Tridacna crocea, 8 for Tridacna maxima, 15 for Tridacna squamosa, and 21 for Tridacna derasa. These seeds were produced from artificially fertilized eggs (see [24] and [25] for details) and were moved into tanks (volume of seawater: 3 to 30 metric tons) at densities of 0.2 to 0.85/ml on Day 1. The number of seeds moved into tanks were estimated from the volume of seawater in the tanks and the density of seeds. Of the 61 production trials the seawater in tanks had been UV treated 23 times in total: 6 times for T. crocea, 6 for T. maxima, 5 for T. squamosa, and 6 for T. derasa. The seeds were provided with either fresh or cultured Symbiodinium three times during Days 3 to 9 at densities of 5 to 30 cells/ml. Approximately each week more than 70% of the seawater was exchanged. The seawater was aerated. On about Day 18 we checked 0.4 to 10 l of the seawater in tanks under a microscope and recorded the number of seeds having attained symbiosis establishment.

### UV Treatment Experiment

The effect of UV treatment of seawater on the symbiosis rate was examined for each of 0.2, 0.7, 1.6, 3.0, 5.0, 11.0, and 20.0-µm filtered seawater with 3 replications: (UV or non UV treatment)×(7 filtration levels) ×3 rearing bottles = 42 bottles in total. On 18 September 2009, eggs of T. crocea were artificially fertilized. On Day 1 the seeds started to be reared at a mean ± SD density of 0.54±0.19/ml in transparent glass bottles (volume: 100 ml), which contained 60 ml of seawater belonging to one of the 14 treatments. Symbiodinium cultured after being extracted from T. crocea mantle were given to the seeds on Day 3 at a density of about 40 cells/ml. No food phytoplankton such as Isochrysis [16] was given to the seeds. On Day 11, half the seawater in each bottle of each treatment was slowly siphoned through a 20-µm mesh with a pipette, then disposed and exchanged with new seawater of the same treatment. The 20-µm mesh prevented both living and dead larvae from being removed. Seawater was agitated every day with a glass stick in each bottle. The water temperature was 28.4±0.9°C (mean ± SD), and the photon energy density was 73.0±24.7 µmol/m^2^/s for 12 h and 0 µmol/m^2^/s for the other 12 h. The photon energy density approximates the level necessary for symbiosis establishment (100 to 200 µmol/m^2^/s) [19] and is needed to prevent various algae (especially Cyanophyceae) from growing in the hatchery tanks [19]. Whether each seed attained symbiosis establishment was checked under a microscope on Days 19 to 20.

### Filtration Autoclave Experiments

Three runs of filtration autoclave experiments were conducted for T. crocea seeds, using a 2-way orthogonal design as follows. Run 1: (0.2, 0.7, 1.6, 3.0, 5.0, 11.0, and 20.0-µm filtration)×(with or without being autoclaved)×(3 rearing bottles) = (42 bottles in total); Runs 2 and 3: (0.2, 3.0, and 20.0-µm filtration)×(with or without being autoclaved)×(3 rearing bottles) = (18 bottles in total). Run 1 was conducted from 18 October 2008 when T. crocea spawned until 03 November 2008 when symbiosis establishment was checked, and Runs 2 and 3 from 19 September to 5 October 2008. In each run the seeds were reared and checked in a similar way to the UV treatment experiment. In Run 3, however, the seawater for rearing was sampled at Ohama (24°20′50′′N, 124°12′01′′E), Ishigaki Island, where the seawater was turbid and this was considered to possibly change the effects of the autoclaved treatment of seawater.

### Monoculture Experiments

Monoculture experiments consisted of two runs. Run 1 was conducted for T. crocea seeds from 17 July 2009 when the clams spawned until 4 August 2009 when the survival/death of the seeds was checked; and Run 2 for T. maxima from 11 to 30 May 2009. In Run 1 seawater near SNFRI was 3-µm filtered and was divided into 3 groups. The first group was UV treated, the second autoclaved, and the third non treated. The seawater of each group was poured into 5 bottles (60 ml), in which T. crocea seeds were reared from Day 1. Rearing conditions (larval density, bottle size, light, temperature, water exchange) were set as in the above experiments, except the seeds were reared without Symbiodinium. Whether each seed was dead or alive was checked under a microscope. In Run 2, T. maxima seeds were reared in 3-µm filtered seawater with or without UV treatment, and the survival/death was checked as in Run 1.

### Feeding Experiments

Two runs of feeding experiments were conducted for T. crocea seeds. Run 1 was conducted during 5 to 22 July 2010, and Run 2 during 22 September to 9 October 2010. In each run T. crocea seeds were reared in a similar manner to the UV treatment experiment in general. In Run 1 we used 48 bottles, adopting a 4-way orthogonal factorial design: 2 sterilization treatments (3-µm filtered seawater with or without UV treatment) ×2 phytoplankton species (Isochrysis sp. or Chaetoceros sp.) ×6 phytoplankton densities (0, 500, 1000, 5000, 10000, or 50000 cells/ml) ×2 feeding rates (phytoplankton given every day or at a 3-day interval) ×1 replication (i.e. 1 bottle). The seawater in each bottle was exchanged with new seawater of the same treatment on Day 7. In Run 2 we used 40 bottles, adopting a 4-way orthogonal factorial design: 2 sterilization treatments (3-µm filtered seawater with or without UV treatment) ×2 antibiotic treatments (0 or 5 mg of streptomycin/l) ×5 phytoplankton densities (0, 10, 100, 1000, or 10000 cells/ml of Isochrysis sp.) ×2 water exchange rates (treated seawater exchanged every day or on only Day 7) ×1 replication. At the end of each run, we checked under a microscope whether each seed in each bottle attained symbiosis establishment.

### Statistical Analyses

Different statistical analyses were performed for the hatchery observations and laboratory experiments. For the hatchery observations, symbiosis rate in a tank was estimated as: (volume of seawater in a tank)/(volume of seawater sampled to check symbiosis establishment on Day ≈18)×(# seeds having attained symbiosis establishment in the sampled seawater)/(# all the seeds moved into the tank on Day 1). It was not possible to simply calculate symbiosis rate as (# seeds having attained symbiosis establishment in the seawater sampled on Day ≈18)/(# all the seeds in the seawater sampled on Day ≈18). This is because some of the seeds having died before the sampling of seawater had degraded or had been washed away during weekly exchange of seawater, which led to an underestimate of the number of dead seeds in the seawater sampled on Day ≈18 and, thus, to an overestimate of the symbiosis rate. The estimated symbiosis rates were compared between UV and non UV treated seawater by Welch’s t test with tanks regarded as replications, using the function “t.test” in R 2.14.0 [26]. This test was performed for each species except T. maxima, data for which was insufficient for statistical tests (only 8 observations).

For the laboratory experiments, symbiosis rate was calculated as (# seeds having attained symbiosis establishment in a bottle)/(# all the seeds in the bottle), and survival rate as (# seeds alive in a bottle)/(# all the seeds in the bottle). Either symbiosis rate or survival rate was analyzed in each run with a mixed-effects logistic regression (MLR, hereafter) [27]. The MLR regarded bottles as a random factor and orthogonally crossed explanatory factors, including all interaction terms. Of the explanatory factors, filtration size and phytoplankton density were analyzed at a ratio scale, whereas the other factors at a nominal scale. Significance probabilities of the explanatory factors were calculated with either likelihood ratio test or F test, depending on whether overdispersion was negligible or not. The MLR was done with the “glmmML” package [28] in R 2.14.0. We present the significance probabilities for only single explanatory factors in Results, unless any interaction term was significant (P<0.05).

## Results

**Figure 1:**
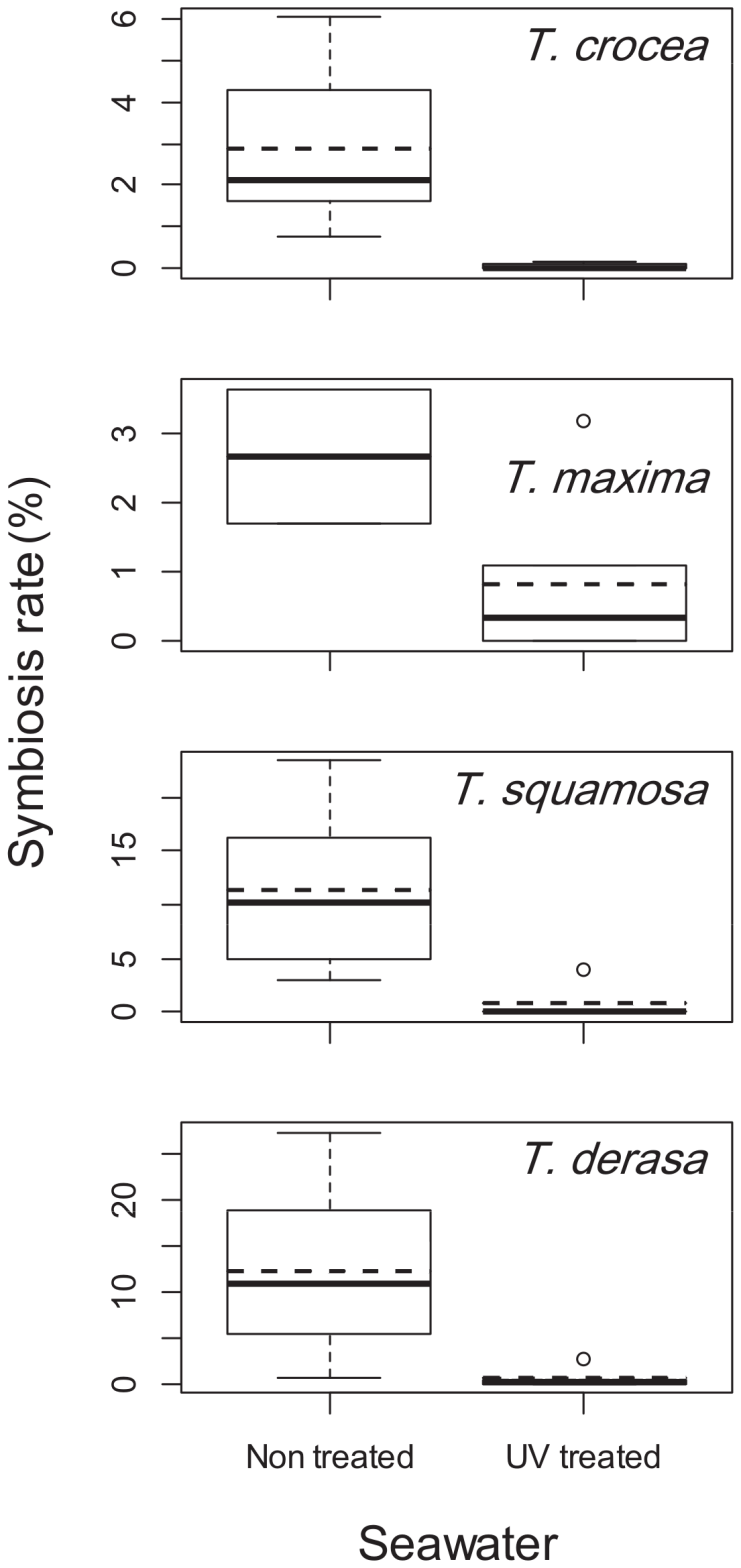
Symbiosis rates of Tridacna spp. in the hatchery observations. Showing what percentage of the seeds reared in non treated and UV treated seawater established a symbiotic relationship with Symbiodinium. Each box plot depicts the mean (dotted bar), the median (thick bar), the upper and lower quartile (upper and lower sides of the box, respectively), the lowest and highest sample within the 1.5 interquartile ranges of the lower and upper quartile (upper and lower tips of the vertical line, respectively), and outliers (circles).

**Figure 2:**
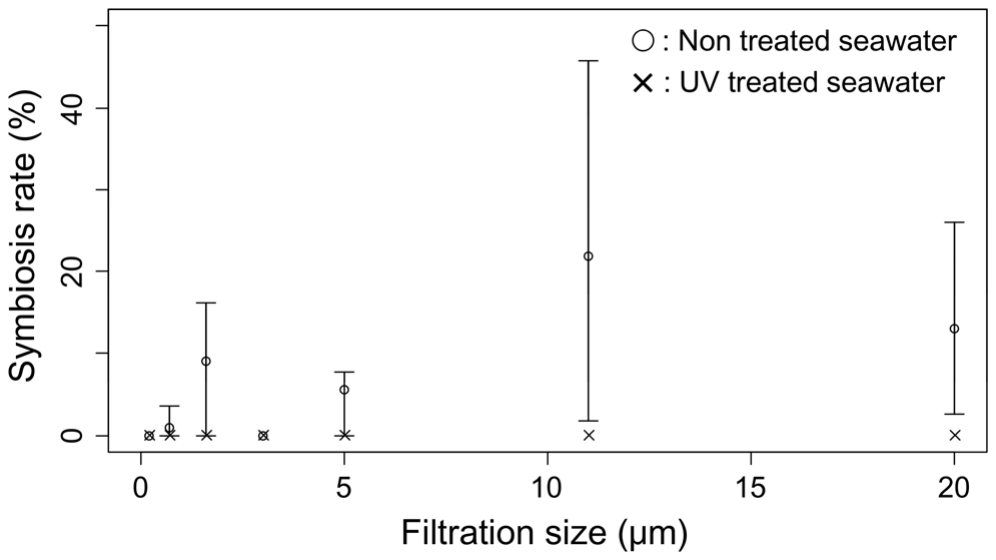
Symbiosis rates of Tridacna crocea in the UV treatment experiment. Showing what percentage of the seeds reared in UV treated and non UV treated seawater filtered at 7 levels established a symbiotic relationship with Symbiodinium. Presented with the mean (circle and cross) and range (vertical line).

In the hatchery observations, symbiosis rates of giant clam seeds were repeatedly found to be far lower for UV treated seawater than for non UV treated seawater. For each species the upper quartile of the symbiosis rate in UV treated seawater was lower than the lower quartile of the symbiosis rate in non UV treated seawater (Figure 1). The mean symbiosis rate was also lower for UV treated seawater (0.0 to 0.8%) than for non UV treated seawater (2.7 to 12.2%) for Tridacna crocea (t = 4.67, df = 10.04, p<0.001), T. maxima (no test), T. squamosa (t = 4.60, df = 11.11, p<0.001), and T. derasa (t = 5.34, df = 15.13, p<0.001). In the UV treatment experiment (Figure 2) symbiosis rates were also significantly lower in UV treated seawater (mean symbiosis rate across rearing bottles and filtration sizes: 0.00%) than in non UV treated seawater (7.82%): F = dev_1_/df_1_/(dev_2_/df_2_) = 17.75/1/(45.51/38) = 14.82, p<0.001 in an F test in MLR.

**Figure 3:**
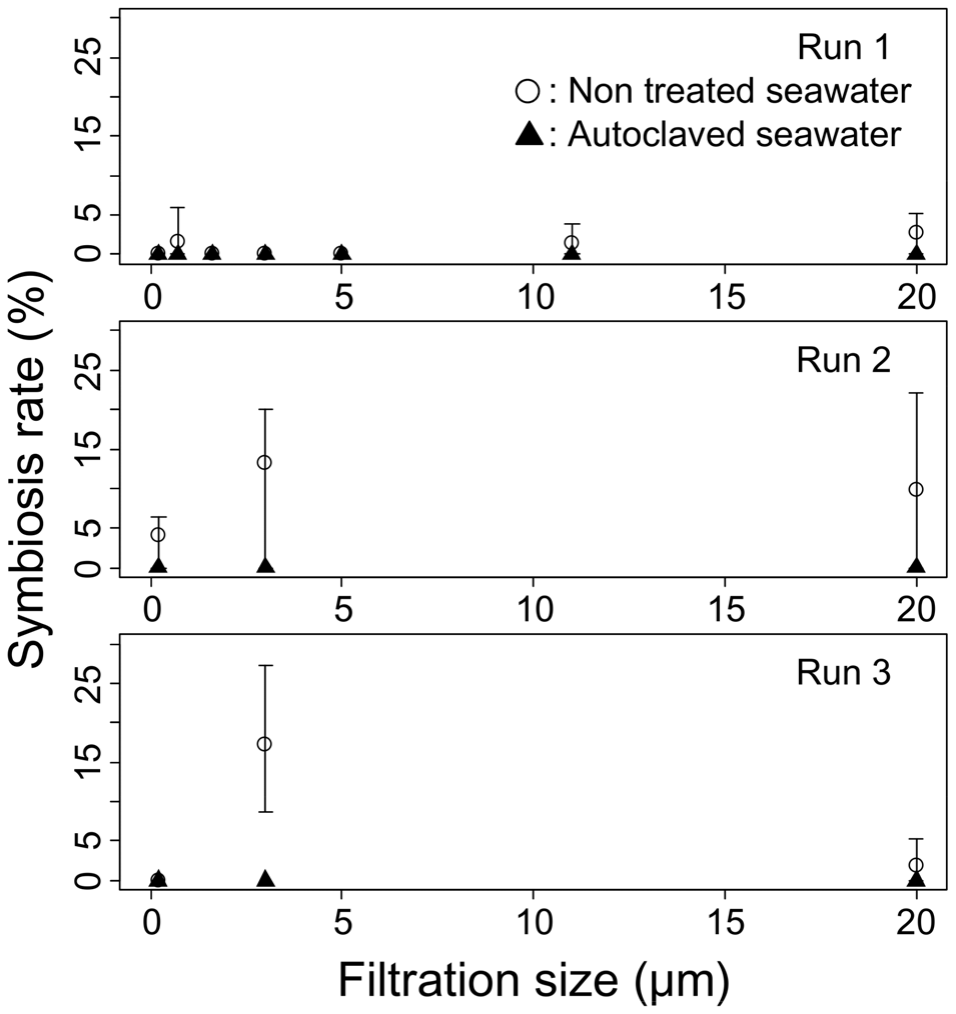
Symbiosis rates of Tridacna crocea in the filtration autoclave experiments. Showing what percentage of the seeds reared in autoclaved and non autoclaved seawater filtered at various levels established a symbiotic relationship with Symbiodinium. Presented with the mean (circle and cross) and range (vertical line).

In the filtration autoclave experiments symbiosis rates decreased for not only UV treated seawater but also for autoclaved and 0.2-µm filtered seawater (Figure 3). In each run symbiosis rates were lower for autoclaved seawater (mean symbiosis rate across rearing bottles and filtration sizes: 0.00% for each run) than for non autoclaved seawater (mean symbiosis rate: 0.84, 8.47, 6.49% for Runs 1, 2, 3, respectively). The difference was significant in MLR for each run: X^2^ = 4.71, df = 1, p = 0.0299 for Run 1; X^2^ = 13.41, df = 1, p<0.001 for Run 2; and X^2^ = 5.77, df = 1, p = 0.0163 for Run 3. In each run mean symbiosis rate for 0.2-µm filtered seawater across the autoclave treatment and bottles (0.00, 2.13, 0.00% for Runs 1, 2, 3, respectively) was lower than for more coarsely filtered seawater (0.55, 4.71, 6.17% for Runs 1, 2, 3, respectively). The effect of filtration size was nearly significant for Run 1 (most likely coefficient ± SE = 0.116±0.064, X^2^ = 3.40, df = 1, p = 0.065 in MLR) but not for Run 2 (most likely coefficient ± SE = 0.019±0.038, X^2^ = 0.235, df = 1, p = 0.628) or Run 3 (most likely coefficient ± SE = −0.063±0.076, X^2^ = 0.688, df = 1, p = 0.407). Although the effect of filtration size was often non-significant in the filtration autoclave experiments, it was significant in the UV treatment experiment (Figure 2): mean symbiosis rate across UV treatments and bottles was 0.00% for 0.2-µm filtered seawater and 4.80% for more coarsely filtered seawater; most likely coefficient ± SE = 0.162±0.068, X^2^ = 6.18, df = 1, p = 0.013.

**Figure 4:**
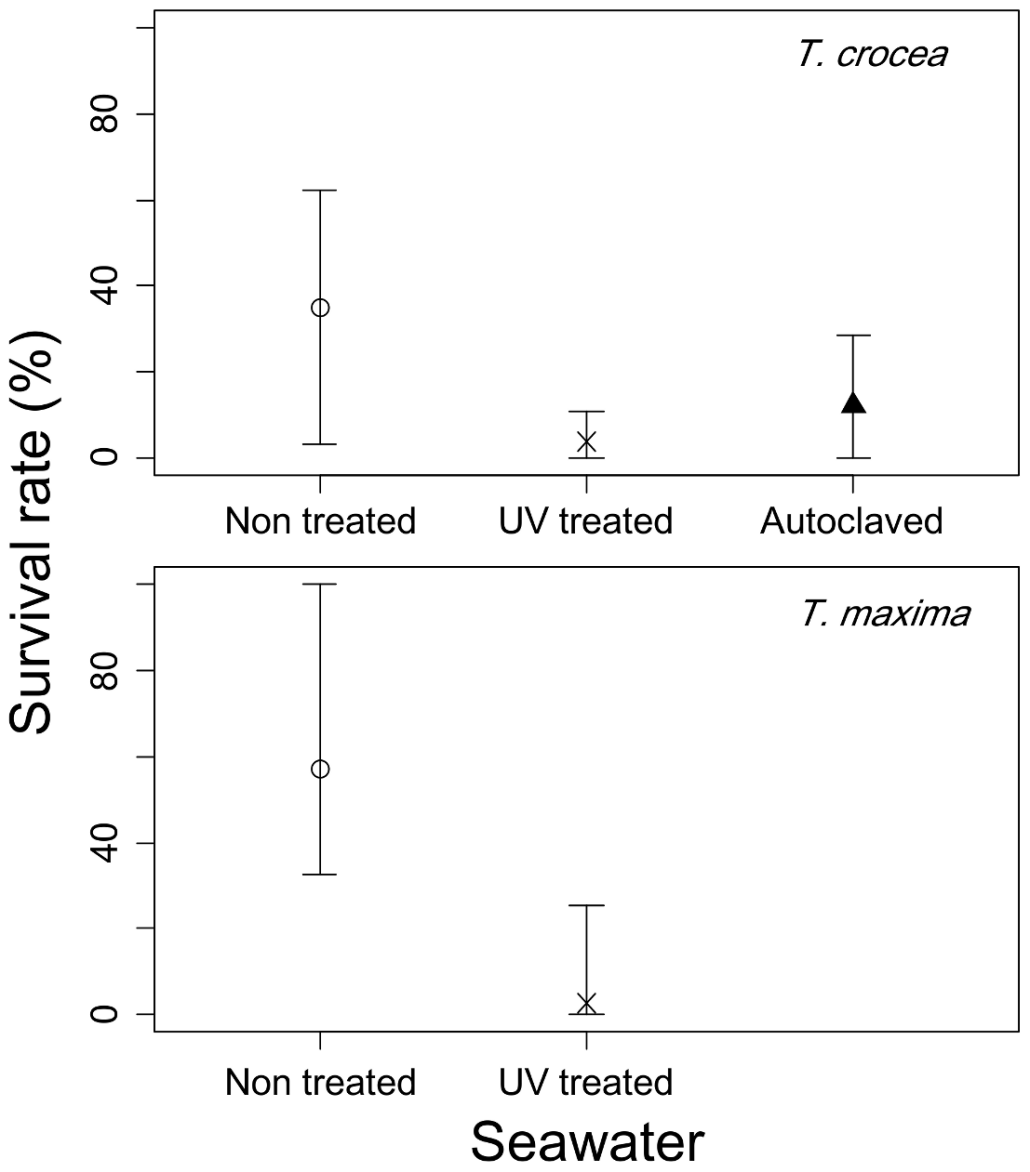
Survival rates of Tridacna crocea (Run 1) and Tridacna maxima (Run 2) in the monoculture experiments. Showing the mean (circle, cross, triangle) and range (vertical line) of survival rates of the seeds reared in non treated, UV treated and autoclaved seawater with no addition of Symbiodinium. For Run 2, autoclaved seawater was not tested.

In the monoculture experiments, giant clam seeds survived in non treated seawater better than in sterilized seawater (Figure 4). T. crocea seeds showed much higher survival rates for non treated seawater (minimum, mean, maximum: 3.4%, 35.1%, 62.2%, respectively) than for UV treated seawater (0.0%, 3.8%, 10.9%) and autoclaved seawater (0.0%, 12.4%, 28.6%), albeit showing no significant difference (MLR: F = dev_1_/df_1_/(dev_2_/df_2_) = 10.97/2/(38.52/11) = 1.57, p = 0.252). Likewise, T. maxima seeds showed much higher survival rates for non treated seawater (minimum, mean, maximum: 32.8%, 57.0%, 100%, respectively) than for UV treated seawater (0.0%, 2.8%, 25.2%; significance probability was not available because MLR did not converge).

In the feeding experiments, giant clam seeds rarely attained symbiosis establishment in UV treated seawater, even if the seeds were fed food phytoplankton in various conditions. In Run 1 (Table 1) T. crocea seeds attained symbiosis establishment in 9 bottles containing non treated seawater but in only 1 bottle (50,000 cells/ml of Isochrysis) containing UV treated seawater. In non treated seawater symbiosis rates irregularly varied among food phytoplankton abundances. Such variation pattern in symbiosis rate was reflected by an MLR which detected a significant effect for UV treatment (X^2^ = 10.91, df = 1, p<0.001; mean symbiosis rate was 0.11% for UV treated seawater and 2.42% for non treated seawater) but not for phytoplankton abundance (most likely ± SE coefficient = 0.00±0.00, X^2^ = 0.69, df = 1, p = 0.40), phytoplankton species (X^2^ = 0.08, df = 1, p = 0.77), or feeding rate (coefficient = −1.07±0.78, X^2^ = 1.85, df = 1, p = 0.173). Likewise, in Run 2 (Table 2) T. crocea seeds attained symbiosis establishment almost exclusively in nontreated seawater regardless of water exchange rate, streptomycin, or phytoplankton abundance: mean symbiosis rate was 0.22% for UV treated seawater and 12.34% for non treated seawater. In non treated seawater the phytoplankton abundance maximizing symbiosis rate varied across combinations of water exchange rate and streptomycin. This complex variation pattern translated into a significant interaction between food abundance, water exchange rate, and phytoplankton abundance in an MLR: X^2^ = 4.27, df = 1, p = 0.039.

**Table 1:** Symbiosis rates of Tridacna crocea in Run 1 of the feeding experiments.

			Non treated seawater		UV treated seawater	
Interval of water exchange	Phytoplankton	Phytoplankton density (cells/ml)	Symbiosis rate	# Seeds observed	Symbiosis rate	# Seeds observed
3 days	Isochrysis sp.	0	0.0%	24	0.0%	44
		500	0.0%	27	0.0%	56
		1000	5.3%	19	0.0%	46
		5000	13.9%	36	0.0%	36
		10000	0.0%	25	0.0%	42
		50000	4.1%	49	2.6%	39
	Chaetoceros sp.	0	0.0%	36	0.0%	31
		500	12.0%	25	0.0%	28
		1000	0.0%	30	0.0%	39
		5000	0.0%	36	0.0%	45
		10000	0.0%	23	0.0%	26
		50000	7.1%	28	0.0%	30
1 day	Isochrysis sp.	0	0.0%	72	0.0%	44
		500	0.0%	36	0.0%	32
		1000	2.5%	40	0.0%	35
		5000	2.8%	36	0.0%	40
		10000	0.0%	29	0.0%	28
		50000	0.0%	25	0.0%	39
	Chaetoceros sp.	0	0.0%	35	0.0%	35
		500	0.0%	23	0.0%	38
		1000	8.3%	36	0.0%	41
		5000	0.0%	38	0.0%	42
		10000	2.9%	35	0.0%	32
		50000	0.0%	23	0.0%	30

Showing what percentage of the seeds established a symbiotic relationship with Symbiodinium after the seeds had been reared in non treated and UV treated seawater, provided with food phytoplankton.

**Table 2:** Symbiosis rates of Tridacna crocea in Run 2 of the feeding experiments.

			Non treated seawater		UV treated seawater	
Interval of water exchange	Streptomycin	Phytoplankton density (cells/ml)	Symbiosis rate	# Seeds observed	Symbiosis rate	# Seeds observed
7 days	No administration	1	11.8%	17	0.0%	33
		10	4.5%	22	0.0%	32
		100	0.0%	25	0.0%	35
		1,000	0.0%	17	0.0%	23
		10,000	0.0%	12	0.0%	26
	Administration	1	0.0%	24	0.0%	32
		10	4.5%	22	0.0%	29
		100	4.0%	25	0.0%	22
		1,000	0.0%	17	4.5%	22
		10,000	26.9%	26	0.0%	37
1 day	No administration	1	13.0%	23	0.0%	18
		10	16.7%	12	0.0%	16
		100	35.3%	17	0.0%	18
		1,000	33.3%	9	0.0%	11
		10,000	0.0%	15	0.0%	22
	Administration	1	0.0%	22	0.0%	17
		10	52.4%	21	0.0%	16
		100	31.3%	16	0.0%	18
		1,000	15.8%	19	0.0%	21
		10,000	10.0%	20	0.0%	16

Showing what percentage of the seeds established a symbiotic relationship with Symbiodinium after the seeds had been reared in non treated and UV treated seawater, provided with food phytoplankton and streptomycin.

## Discussion

The hatchery observations, UV treatment experiment, and feeding experiments demonstrated that symbiosis rates of giant clam seeds (Tridacna crocea, T. maxima, T. squamosa, T. derasa) sharply decreased when the seeds were reared with Symbiodinium in UV treated seawater after fertilization. These results confirm the reproducibility of the adverse effect of UV treatment suggested previously [20]. The results also accord with previous studies. To our knowledge, only Belda-Baillie et al. [11] reported successful symbiosis establishment (symbiosis rate >0.6% to 2.6%) for giant clam seeds that had been constantly reared in UV treated seawater after fertilization. In contrast, many researchers, e.g. [12], [18], [29], [30], [31], reported successful symbiosis establishment for the seeds reared in seawater that appears to be non UV treated (these researchers did not state that they treated seawater with UV). In addition, Ellis [16] and Mingoa-Licuanan and Gomez [15] recommend rearing giant clam juveniles in non UV treated seawater. Overall, rearing giant clam seeds with Symbiodinium constantly in UV treated seawater leads to a decreased symbiosis rate.

The decreased symbiosis rates are unlikely to be due to ozone, which arises in UV treated seawater and inhibits normal development of marine animals [32], [33]. This is because the UV treated seawater was left for ≈ 1 day before the start of larval rearing in our study and showed similar ozone levels with non UV treated seawater.

In contrast, the mechanism causing the reduced symbiosis rates for UV treated seawater is possibly identical to the mechanism for other sterilized seawater, namely, 0.2-µm filtered and autoclaved seawater. This is indicated from our filtration autoclave experiments in which symbiosis rates decreased for autoclaved seawater and 0.2-µm filtered seawater as well as UV treated seawater. Symbiosis rates averaged across our observations and experiments were only 0.2% for seawater sterilized by at least one of UV treatment, autoclave or 0.2-µm filtration but 7.2% for unsterilized seawater (Table S4). Between these two values (0.2 and 7.2%) is a previously reported symbiosis rate, 0.3% (averaged across two runs of experiments by [19] on T. crocea at temperatures between 25 to 32°C; see Figure 1 in [19]). Inhibition of normal development due to sterilization of seawater has also been reported for coral polyps [34], [35], [36].

The possible mechanisms causing reduced symbiosis rates include such mechanism that giant clam seeds and/or Symbiodinium are damaged by sterilization of seawater. Of the two taxa, giant clam seeds at least are likely to be damaged owing to sterilization of seawater. This is indicated from our monoculture experiments: giant clam seeds reared without Symbiodinium showed lower survival rates in sterilized seawater than in non treated seawater. In addition, survival rates, which were calibrated as a value per 14 days and averaged across all of our experiments, were only 16.4% for seawater sterilized by at least one of UV treatment, autoclave or 0.2-µm filtration but 37.3% for unsterilized seawater (Table S5). Between these two values (16.4 and 37.3%) are previously reported values: 16.6% for Hippopus hippopus (see Figure 2 of [37]) and 32.4% for Tridacna squamosa (see Figure 5 of [10]). What are the possible adverse effects of sterilization of seawater on giant clam seeds? In our feeding experiments, giant clam seeds rarely established a symbiotic relationship with Symbiodinium in sterilized seawater, even if the seeds were provided with living food phytoplankton, Isochrysis or Chaetoceros, in various rearing conditions. Hence, such food phytoplankton is unlikely to be related with the possible adverse effects of sterilization of seawater. This is also indicated from our UV treatment experiment and filtration autoclave experiments: the symbiosis rates for 3-µm filtered seawater, which would contain few large food phytoplankton (e.g. Chaetoceros, Isochrysis, Pavlova, Tetraselmis, and Dunaliella; the longest part of cell>about 3 µm) [38], were similar to those for 5 to 20-µm filtered seawater, which would contain the phytoplankton more abundantly.

Although large food phytoplankton are not linked to the possible adverse effects of sterilization of seawater on giant clam seeds, there remain many hypotheses on the adverse effects of sterilization of seawater. One of the hypotheses is associated with small plankton (<3 µm) in the seawater. Our experiments mainly used 3-µm filtered seawater sampled from the subtropical sea, which included at least 37.2 to 56.6% of the total weight of particle suspension in the seawater. This particle suspension, if unsterilized, is likely to include many live picoplankton (0.2–2 µm) and nanoplankton (2–20 µm) [39], [40]. These small plankton are likely to be important food items for coral reef organisms. For example, they are reported to account for >90% of the nitrogen removed from water column particulates by coral reef communities [40]. These small plankton are also likely to be important food items for giant clam seeds as reported for other bivalves [41]. These small plankton have high rates of production and appear to be continuously consumed by higher trophic level organisms [39]. Therefore, it is possible that seawater sterilization would stop the production of the small plankton, thereby removing the food supply to giant clam seeds, which translates into reduced survival and symbiosis rates. Another hypothesis on the adverse effects of seawater sterilization is that sterilization leads to a decrease of the bacteria that decompose substances being harmful to giant clam seeds (e.g. ammonia; see [42] for bacterial decomposition of ammonia). A third hypothesis is that seawater sterilization results in decreased microbial diversity, which, paradoxically, translates into an increase of harmful bacteria that can adhere to giant clam seeds when the seeds are artificially hatched in a tank ([15], [24]). Degradation of the ecosystem function due to decreased biodiversity is reported in [43] and [44].

Seawater sterilization might affect not only giant clam seeds, as mentioned above, but also Symbiodinium. A hypothesis is that Symbiodinium requires some bacteria-produced substances, and thus sterilization of seawater reduces the production rate of Symbiodinium, and thereby, the symbiosis rate. Many species of marine algae need vitamin B12, which symbiotic bacteria produce [45]. Conversely, another hypothesis is that some bacteria lead to a deterioration of seawater through, for example, depriving Symbiodinium of nutrients, which leads to an increase of the relative benefit of the environment inside the giant clam body and enhance symbiosis establishment. Coexistence of species often occurs in harsh environments rather than in mild environments [46]. Such harsh environment for Symbiodinium might be lost owing to seawater sterilization.

### Concluding Remarks

Although the mechanism inhibiting symbiosis establishment in sterilized seawater remains unclear, symbiosis rate was found to decrease when giant clam seeds were continuously reared in seawater sterilized by use of UV, autoclave, or 0.2 µm filter. Such a decrease was repeatedly found for UV treated seawater in both the hatchery and the laboratory. Therefore, it seems better for seed producers to avoid continuous rearing of giant clam seeds in sterilized seawater, especially UV treated seawater.

## Supporting Information

Table S1 Ozone density of UV and non UV treated seawater: Ozone density was measured for UV treated and non UV treated seawater with an Eutech C105 O-zone Colorimeter (http://www.eutechinst.com/pdt-type-colorimeters-C401.html). In the hatchery, seawater was poured into a tank after UV treatment and also into another tank without UV treatment on 15 November 2009. One day later, the ozone density of these seawater samples was measured. In the laboratory, seawater was filtered through 0.2 µm and 20 µm filters, and a half of each of the filtered seawater was UV treated and another half non UV treated on 27 January 2010. One day later the ozone density of the four samples, (0.2- or 20-µm filtered)×(UV− or non treated), was measured.
(XLS)

Table S2 Bacterial densities for filtered seawater: Bacterial density was estimated for the seawater that was sampled from the sea surface near SNFRI. On 02 May 2009 sampled seawater was divided into 3 groups. One of the groups was UV treated, another autoclaved, and the last one not treated. Aliquots of each group were spread onto 3 agarose plates, and the bacterial colonies thereon were counted 3 days later to estimate the bacterial density (see [24] for the detailed procedure). On 24 January and 4 December 2010 seawater was again sampled and divided into 7 groups, each of which then was filtered through 1 of 7 filters (0.2, 0.7, 1.6, 3, 5, 11, or 20 µm). For each of the 7 filtered seawaters the bacterial density was estimated again.
(XLS)

Table S3 Particle suspension density: The weight of particulates in seawater was measured for 7 fractions (0.2 to 0.7, 0.7 to 1.6, 1.6 to 3, 3 to 5, 5 to 11, 11 to 20, and >20 µm) 3 times: 11 May 2009, 19 May 2010, and 29 November 2010. On each day seawater was sampled and filtered in the order of 20, 11, 5, 3, 1.6, 0.7, and 0.2 µm filter. The filters used were dried at 60°C for 24 hours and the increase of dry weight of each filter was divided by the volume of seawater passing through the filter to calculate the particle suspension density.
(XLS)

Table S4 Mean symbiosis rates for unsterilized and sterilized seawater.
(XLS)

Table S5 Mean survival rates for unsterilized and sterilized seawater.
(XLS)
